# The effect of heracleum persicum (Golpar) oil and alcoholic extracts on sperm parameters and chromatin quality in mice

**Published:** 2016-06

**Authors:** Neda Taghizabet, Esmat Mangoli, Fatemeh Anbari, Seyed Ali Masoodi, Ali Reza Talebi, Malihe Mazrooei

**Affiliations:** 1 *Department of Biology and Anatomical Sciences, Shahid Sadoughi University of Medical Sciences, Yazd, Iran.*; 2 *Research and Clinical Center for Infertility, Shahid Sadoughi University of Medical Sciences, Yazd, Iran.*; 3 *School of Medicine, Shahid Sadoughi University of Medical Sciences, Yazd, Iran.*; 4 *School of Health, Shahid Sadoughi University of Medical Sciences, Yazd, Iran.*

**Keywords:** *Heracleum persicum extracts*, *Sperm*, *Mice*, *Chromatin*, *DNA integrity*

## Abstract

**Background:**

Evaluating the significance and the effects of plant-derived drugs on laboratory animal’s fertility was recognized. There was antioxidant activity reported from Heracleum persicum (Golpar).

**Objective:**

Current study aims to study the antioxidant effect of Golpar extracts on sperm parameters and chromatin quality in mice.

**Materials and Methods:**

Eighteen adult male mice were divided to 3 groups (10 wk old, 35 gr weight): group1 received hydro alcoholic extract (1000 mg/kg, ip), group 2 received oil extract (200 mg/kg, ip) and group 3 serving as the sham control group that received sterile water. Finally, left cauda epididymis of each animal was dissected and sperm analysis was done accordingly. To asses sperm chromatin and DNA quality, we used aniline blue (AB), toluidine blue (TB), chromomycin A3 (CMA3) and acridine orange (AO) staining.

**Results:**

Progressive and non-progressive sperm motility were significantly increased in group 1 in comparison with group 3 (p=0.032). There was an increasing trend in progressive sperm motility and decreasing trend in non-progressive sperm motility in group 2 in comparison with group 3, but the differences were not significant (p=0.221 and p=0.144, respectively). According to the sperm chromatin quality, the results of TB and AO tests revealed significant differences (p=0.004, p=0.000, respectively) between those groups and showed that the extracts of Golpar cause DNA damage, but no differences can be observed between them in AB and CMA3 staining (p>0.05).

**Conclusion:**

The results showed that Heracleum persicum extracts may improve sperm motility. Also, it has harmful effects on sperm chromatin condensation and DNA integrity in mice.

## Introduction

Oxidative stress (OS) is a real item in reproductive tract based on potential and harmful impacts on high level of reactive oxygen species (ROS) on sperm’s number, motility, quality, and function which includes some damage to sperm nuclear DNA (1). Antioxidants are important substances, which are able to protect body from damages of free radical-induced oxidative stress. There are different free radical scavenging antioxidants in body, which many of them are derived from dietary sources like fruits, vegetables, and teas (2). Innumerable studies reported lucrative effects of antioxidant drugs on semen quality, but no well-defined therapeutic protocol can be seen in male infertility (3). Therapeutic effects of some medical plants and vegetables are well known since they generally are used as food and folk medicine for many diseases as well. Antioxidant capacities of Heracleum persicum (H.persicum), is a flowering plant from Apiaceae family, were evaluated by determining their effects on 2,2-diphenyl-1-picrylhydrazyl (DPPH )radical scavenging, and lipid peroxidation inhibition, as well as their total phenolic contents (4). Moreover, some antioxidant of glutathione category has also been seen in this plant (5). Hydro alcoholic, an extractive of H.persicum include several furanocoumarins (6).

Recently, some researches have revealed several benefits of H.persicum. Herbal medicine observations have shown some antioxidant, anticonvulsant, analgesic, anti-inflammatory, immune modulatory and cytotoxic effects (6). In traditional medicine it was observed that use of H.persicum during the sexual cycle stops progression of ovarian phase in females (7).

Dalouchi *et al* showed that the extract of H.persicum in association with cyclo phosphamide significantly improved sperm parameters (8)., Seminal plasma of infertile men has worse antioxidant levels in comparison with fertile men, especially those who have poor sperm motility. The existence of ROS activity in infertile groups sperm is along with much lower levels of chain-breaking antioxidants in seminal plasma as well (9). It seems that the role of these biological antioxidants and their related mechanisms is the exact significant area for further studies in infertility treatment (10).

One of the microscopic assessments of sperm for male fertility investigation is the evaluation of sperm nuclear chromatin; any anomalies in sperm chromatin can affect embryonic development (11). It is usually believed that there is a clear relationship between sperm chromatin/DNA damage and reproductive outcomes. Furthermore, sperm chromatin condensation has a key role in male fertility, early embryonic growth and pregnancy results. In spermatogenesis process , sperm chromatin compaction degree changes intensely when histones are replaced in a stride mode by testis-specific nuclear proteins, transitional proteins and finally by protamine. 

Inter- and intra-molecular disulphide bonds of protamine molecules are crucial for sperm nuclear compaction and stabilization. Each anomaly during this process may cause male infertility (12). There are some kinds of tests for sperm chromatin/DNA evaluation which demonstrate different forms of damages. Chromatin structural probes by nuclear dyes with cytochemical bases are sensitive, easy and inexpensive which do not need unique tool like flow cytometry (13). 

In this paper we aim to investigate the effect of *Heracleum persicum *Desf ex Fischer (*umbelliferae*) on sperm parameters and chromatin quality in mice.

## Materials and methods

**Animals and treatment**

In this experimental study, 18 adult male mice (10 wk old, 35 gr) were divided to 3 groups: group 1 received hydro alcoholic extract (1000 mg/kg, ip), group 2 received oil extract (200 ml/kg, ip), and group 3 serving as the sham control group that received sterile water (16(. The mice were held in separate cages about 35 days (duration of spermatogenesis) and were housed in a controlled environment with a temperature ranged 25±2^o^C and mean relative humidity of 50±5%. They were fed “mice chow” and had access to water ad libitum. The experimental project was approved by ethics committee of Shahid Sadoughi University of Medical Sciences, Yazd, Iran. 

**Plant material and preparation of hydro alcoholic extract and essential oil**

Fruits of H.persicum were collected from Yooush Village , mazandaran, Iran. The plant identity was confirmed by the Botany Department of Yasuj University. A voucher specimen (No. 34355) was deposited in Yasuj Herbarium, Yasuj University of Biology Sciences, Yasuj, Iran for further reference. Alcoholic extract of H.persicum was used because of its useful information about the antioxidant activity and flavonoid content. H.persicum's alcoholic extract was partitioned successively and compared with oil extract (14). 

For preparation of hydro alcoholic extract, air-dried and powdered fruits of the plant (200 gr) were macerated with 1500 ml of EtOH-H_2_O (7:3) for 48 hr. Then the extract was shacked, filtered, and evaporated in rotating evaporator under reduced pressure until dryness (15). Evaporation and solvent removal of hydro alcoholic extract gave semi-solid masses (yield 7.5%). The essential oil was isolated by powdered hydro distillation fruits of plant for 3 hr. Yield value of essential oil was 3.8% (v/w). Essential oil was kept in a cool and dry place and the hydro alcoholic extract was kept in refrigerator (16(.

**Epididymal sperm preparation**

Animals were anesthetized by ketamine and xylazine (150 mg/kg and 10 mg/kg, respectively) and their dissected cauda epididymis were placed in 1 ml of pre-warmed Ham’s F10 medium for 30 min.Gentle tearing was done to swim-out spermatozoa into culture medium (17).

**Sperm analysis**

Sperm parameters including count (10^6^/ml), motility, viability and normal morphology (%) were evaluated for 200 spermatozoa of each animal. Sperm count and motility was assessed using Makler chamber (Sefi Medical Co., Haifa) and light microscopy (Olympus Co., Tokyo, Japan). Briefly, 5 μL volume of sperm suspension was loaded into chamber and the number of sperm counted from 10 squares columnar or horizontal and reported as million sperm/ML. Motility was expressed as percentage of progressive and non-progressive spermatozoa. Sperm viability and morphology were evaluated by Eosin and Papanicolaou staining test respectively (13).

**Sperm chromatin/ DNA integrity assessments**

DNA integrity and chromatin condensation assessments were done by standard cytochemical techniques including AO, AB, TB and CMA3 staining. All of dyes and chemicals were purchased from Sigma Aldrich Company (St Louis, MO, USA). The effectiveness of dyes was tested with and without acid denaturation of some sperm specimens and they were considered as positive and negative controls, respectively (18).

**Aniline blue (AB) staining**

AB selectively stains sperms with residual histones. Briefly, air-dried smears from each sample were fixed in 3% buffered glutaraldehyde in 0.2 M phosphate buffer (pH=7.2) for 30 min at room temperature. Each smear was stained with 5% aqueous AB stain in 4% acetic acid (pH=3.5) for 7 min. In light microscopic evaluation, 200 spermatozoa were counted in different areas of each slide using 100× eyepiece magnifications. Unstained or pale blue stained (normal spermatozoa or AB^-^) and dark blue stained (abnormal spermatozoa, AB^+^) cells were reported as percentage (17, 19).

**Toluidine blue (TB) staining**

TB is a metachromatic dye which determines both quality and quantity of sperm nuclear chromatin condensation. Sperm smears were fixed in fresh ethanol (96%) and acetone (1:1) at 4^o^C for 30 min and then incubated in 0.1N hydrochloric acid at 4^o^C for 5 min. The slides were washed 3 times with distilled water for 2 min and finally stained with 0.05% TB in 50% citrate phosphate for 10 min at room temperature. 

In each sample, at least 200 spermatozoa were counted under light microscopy using 100× eyepiece magnification and according to metachromatic staining of sperm heads were classified in following scores: 0: light blue (good chromatin); 1: dark blue (mild abnormal chromatin); and 2: violet and purple (sever chromatin abnormality). The spermatozoa with score 0 were considered as normal cells (TB^-^) and spermatozoa with violet and purple heads (scores 1, 2) were considered as abnormal cells (TB^+^) (20).

**Acridine orange test (AOT)**

AO is a metachromatic fluorescence staining that is used to determine the rate of DNA denaturation. Briefly, the smears were fixed in Carnoy’s solution (methanol/glacial acetic acid, 3:1) at 4^o^C for at least 2 hr. Each sample was stained by freshly prepared AO (0.19 mg/ml) in McIlvain phosphate-citrate buffer (pH=4) for 10 min. Smears were assessed on the same day using fluorescent microscope (Zeiss Co., Jena, Germany) with a 460-nm filter. At least 200 spermatozoa were evaluated and the rate of normal (green fluorescent) and abnormal cells (red fluorescent) were reported as percentage (19).

**Chromomycin A3 staining**

CMA3 was used to estimate the degree of sperm chromatin protamination. For this staining, the smears were dried and fixed in Carnoy’s solution at 4^o^C for 10 min. The slides were treated with 150 μl of CMA3 (0.25 mg/ml) in McIlvain buffer for 20 min. After staining in darkroom, the slides were washed in buffer and mounted with buffered glycerol. In each sample, at least 200 spermatozoa were counted under fluorescent microscope with a 460-nm filter and 100× eyepiece magnification and the percentage of CMA3^+^ spermatozoa was reported. Bright yellow-stained chromomycin-reacted spermatozoa (CMA3+) were considered as abnormal and yellowish green-stained no reacted spermatozoa (CMA3-) were considered as sperm with normal protamination (20).

**Statistical analysis**

Data are shown as mean±SD. Statistical analysis was performed by SPSS 18 for Windows (SPSS Inc., Chicago, IL, USA). One way ANOVA was used to evaluate the significant differences between 3 groups and the Tukey-HSD post-test was operated for determination of differences between each two groups. The term ‘statistically significant’ was used to signify a two-sided p<0.05 for sperm parameters and cytochemical tests.

## Results

Table I shows the sperm parameters in 3 groups. This table reveals that only progressive and non-progressive motility had significant differences between 3 groups (p<0.05). But there were not any significant differences between alcoholic extract and oil extract group. 

Table II shows the sperm chromatin condensation analysis results and DNA integrity status using different assays. There were statistically significant differences between 3 groups regarding to TB, and AO tests (p<0.001). Also regarding TB test there are significant differences between alcoholic extract and oil extract group (p=0.036) and between oil extract and control group (p=0.003). In addition, regarding AO test, there were statistically significant differences between each two groups (p<0.001). Data showed that the rates of TB+ and AO+ spermatozoa were significantly increased compare with control group. But, in CMA3 and AB staining there were no significant differences between them.

**Table I:** The results of sperm parameters between control and case groups (mean±SD) p-value <0.05

**Variable**	**Alcoholic extract group (1)**	**Oil extract group (2)**	**Control group (3)**	**p-value**
Sperm count (×10^6^)	19.1 ± 7.79	20.66 ± 4.32	23.4 ± 3.98	0.867^a^ 0.374^b^ 0.666^c^ 0.399**d**
Progressive motility (%)	42.6 ± 13.4	36.3 ± 9.22	26.2 ± 6.17	0.536^a^ 0.032^b^ 0.221^c^ 0.038d
Non progressive motility (%)	20.4 ± 8.8	23.6 ± 6.08	31.83 ± 5.7	0.696^a^ 0.032^b^ 0.144^c^ 0.035d
Immotile sperm (%)	37.6 ± 9.55	38 ± 9.46	42 ± 5.29	0.977^a^ 0.565^b^ 0.690^c^ 0.560d
Viability (%)	65.4 ± 10.14	63.6 ± 7.73	60.5 ± 3.57	0.910^a^ 0.510^b^ 0.758^c^ 0.533d
Normal morphology (%)	13.2 ± 3.72	13 ± 3.74	15.6 ± 2.9	0.985^a^ 0.451^b^ 0.366^c^ 0.337**d**

a . Difference between alcoholic extract and oil extract group.

b . Difference between alcoholic extract and control group.

c . Difference between oil extract and control group.

e . Difference between 3 group

**Table II:** The results of sperm chromatin/ DNA evaluation between control and case groups (mean±SD) p-value<0.05

**Variable**	**Alcoholic extract group (1)**	**Oil extract group (2)**	**Control group (3)**	**p-value**
Anline blue (AB) (%)	16 ± 4.1	11.8 ± 2.5	14.3 ± 1.86	0.066^a^ 0.600^b^ 0.332^c^ 0.078^d^
Toluidine blue (TB) (%)	17.1 ± 5.3	24.8 ± 5.1	13.8 ± 3.81	0.036^a^ 0.469^b^ 0.003^c^ 0.004^d^
Acridine orange (AO) (%)	83 ± 9.09	67 ± 5.2	4.6 ± 2.3	0.001^a^ 0.000^b^ 0.000^c^ 0.000^d^
ChromomycinA3 (CMA3) (%)	3.83 ± 1.47	3.18 ± 0.75	4 ± 1.09	0.583^a^ 0.966^b^ 0.438^c^ 0.431^d^

a . Difference between alcoholic extract and oil extract group.

b . Difference between alcoholic extract and control group.

c . Difference between oil extract and control group.

d . Difference between 3 group.

## Discussion

Present study findings showed that H.persicum has beneficial effect on sperm’s progressive and non-progressive motility. In line with our results there was a study that showed sperm motility in experimental groups that received cyclophosphamide along with H.persicum increased which could be attributed to an increase in antioxidant capacity and protective effect of H.persicum*, *(8). 

The sperm plasma membrane has polyunsaturated fatty acids, that are sensitive to peroxidative damage. The lipid peroxidation defeats the creation of the lipid in the spermatozoa membranes and causes axonemal damage and reduce the sperm’s motility, decrease in sperm viability and increase in mid-piece morphological defects. Furthermore, it can even finally stop spermatogenesis in severe cases (8). Antioxidants are compounds that give the ROS control and lipid peroxidation. Most of plants rich in antioxidants have some trend to increase sperm count, motility, and normal morphology. Antioxidants increase sperm condensation that a balance of the benefits and risks from ROS and antioxidants seems to be essential for the spermatozoa survival and activity (21). There are six furano coumarins and flavonoids within the fruits contain H.persicum that have antioxidant functions and can protect sperm from lipid peroxidation(16).

Barzegari Firouzabadi *et al* in their study indicated that consumption of Golpar methanol extract may reduce plasma testosterone, body weight, testis weight and sperm concentration and thus can be used to treat sexual dysfunction in males (22). Difference in the results of these studies with our results could be due to an insufficient dose or duration of their treatment. Several studies showed some benefits of an increased intake of antioxidants by men who show high levels of peroxidation of lipid and fragmentation of sperm which could result in gestational outcomes improvement in couples with history of recurrent embryo losses (23). Structural sperm characteristics were developed as well as improved chromatin integrity after treatment with antioxidant (24). But in current study, using H.persicum as an antioxidant caused bad effects on chromatin condensation and increases DNA denaturation in mice based on TB and AO tests respectively. There were no studies that assess the effect of H.persicum on sperm DNA integrity, so cytochemical assay has been used for the first time to study this. 

A notable difference between groups can be seen in AO test. As the AO test has the potential to differentiate the single-stranded DNA from double-strand ones, it can be concluded the H.persicum extracts increased the denaturation of sperm DNA strands. Regarding to TB staining a significant difference has been found between groups. This revealed that H.persicum extracts causes changes in both the quality and the quantity of nuclear chromatin condensation and increases the sperm DNA fragmentation. No similar study in literature has been found to compare our data with others. In AB and CMA3 staining that shows the sperm cells with excessive histones and protamine deficiency respectively, no significant difference was found between groups. 

Therefore, it can be said that the H.persicum extracts does not have any detrimental effects on histone-protamines replacement during the testicular phase of sperm chromatin packaging. H.persicum contains copper and some chemical compositions such as Hexyl butyrate, Octylacetate, hexyl methyl butanoate and hexylisobuty rate (8). Medina *et al* showed that butyrate enhanced over 7-fold the activation of caspase-3 induced by the addition of cytochrome C and dATP to isolated cytosol (25). 

Perhaps it can be concluded that the presence of this compound in H.persicum extracts cause sperm DNA fragmentation in mice. But, further studies are required to illuminate the mechanism of action and the components in charge of these plant and antioxidant effects.

## Conclusion

Finally, our results showed that H.persicum extracts caused some improvement in sperm motility but it has harmful effects on sperm chromatin condensation and DNA integrity in mice.
